# Study of Peripheral Blood T-Lymphocytes and Their Subpopulations in COVID-19 Patients

**DOI:** 10.1155/bri/5791950

**Published:** 2025-09-15

**Authors:** Huaitai Lin, Jingxing Yi, Die Huang, Jichao Wu, Jun Yin

**Affiliations:** ^1^Department of Orthopedics, Cancer Hospital of Shantou University Medical College, Shantou, Guangdong, China; ^2^Department of Clinical Laboratory Medicine and Division of Hematology, the Second Affiliated Hospital of Shantou University Medical College, Shantou, Guangdong, China

## Abstract

**Objective:** To study the characteristic changes in peripheral blood T-lymphocytes and their subpopulations in COVID-19 patients by in-depth characterization of peripheral blood T-lymphocytes and to analyze the changes in the severity of COVID-19 and age factors in relation to changes in T-lymphocytes and their subpopulations.

**Methods:** T-lymphocytes (including Th cells, regulatory T-lymphocytes, CD4+ initial T-lymphocytes, CD4+ memory T-lymphocytes, CD4+ T-lymphocytes functional subsets, Tc cells, CD8+ initial T-lymphocytes, CD8+ memory T-lymphocytes, and CD8+ T-lymphocyte functional subpopulations) were isolated, by flow cytometry, from peripheral blood specimens of 60 COVID-19 patients (experimental group) and 36 healthy controls (control group). The results of the two groups were compared and further analyzed in relation to age and disease severity of COVID-19 patients.

**Results:** The absolute counts of T-lymphocytes and their subpopulations in the peripheral blood of COVID-19 patients were reduced, and the proportions of the cells in each subpopulation were imbalanced, with the absolute counts of T-lymphocytes and their subpopulations in peripheral blood of critically ill patients being lower relative to those of mildly ill patients, and the absolute counts of CD8+ initial T-lymphocytes in the peripheral blood of COVID-19 patients group being negatively correlated with the age of the patients.

**Conclusion:** SARS-CoV-2 infection has an inhibitory effect on the number of T-lymphocytes and the proportion of their subpopulations, especially in critically ill and elderly patients, suggesting that SARS-CoV-2 infection has a serious impact on the cellular immune function of the body, and that in-depth characterization of T-lymphocyte population can more accurately reflect the changes in immune function to assess the state of cellular immune function.

## 1. Introduction

Coronavirus disease 2019, the new coronavirus pneumonia, commonly referred to as COVID-19, is a respiratory infectious disease caused by infection with a novel coronavirus, denoted as severe acute respiratory syndrome coronavirus 2 (SARS-CoV-2) [1]. Since the end of 2019, COVID-19 has been a global public health problem, which is extremely dangerous and seriously affects people's health and life [2]. Cellular immunity plays an important role in the immune response to viral infection and is critical for the body's clearance of SARS-CoV-2 [3]. The body's cytotoxic T-lymphocytes (Tc cells) clear the virus by secreting a variety of molecules such as interferon, granzyme and perforin, while helper T-lymphocytes (Th cells) play a role by synergizing with killer T cells and B lymphocytes. It has been reported in the literature that T-lymphocytes are rapidly and markedly reduced in the peripheral blood of patients infected with the SARS virus in the acute phase of the infection, in contrast to the proliferative lymphocyte response that occurs following infection with HIV-1, CMV, or EBV [4, 5]. T-lymphocytes have been found to be susceptible to MERS-CoV, which also causes massive apoptosis of T-lymphocytes by infecting human peripheral blood mononuclear cells, lymphoid organs such as tonsils and spleen, as well as CD4+ T-lymphocytes and CD8+ T-lymphocytes in marmoset spleens [6]. Several studies have shown that T-lymphocytes are reduced in patients with COVID-19 compared to healthy individuals [7–9]. A decrease in T-lymphocytes has been observed in patients with COVID-19 pneumonia compared to healthy individuals, along with the number of peripheral blood lymphocytes, monocytes, and granulocytes, including CD4+ T-lymphocytes and CD8+ T-lymphocytes, and the CD4+/CD8+ ratio [10, 11], although some studies have shown insignificant differences in CD4+/CD8+ ratios [12]. In 2020, Chao-Lin Huang and colleagues conducted a study on 41 cases of COVID-19, diagnosed at the Wuhan Jinyintan Hospital, and found that 26 out of 41 patients had lymphocytopenia [13]. Follow-up studies have also shown that leukocytes, total lymphocytes, CD4+ T and CD8+ T cells, and the CD4+/CD8+ T-lymphocyte ratio in patients with COVID-19 are decreased compared to normal subjects [14]. Studies have found that the counts and percentages of CD4+ T and CD8+ T-lymphocytes correlate with the severity of COVID-19 and are predictive of COVID-19 disease severity in patients [15, 16]. Shin et al. showed that CD8+ T-lymphocyte counts are lower in MERS patients than in healthy subjects and correlate with disease severity [17]. A United Kingdom study of patients with hematologic neoplasms in combination with COVID-19 showed that patients with high CD8+ T-lymphocyte counts also had higher survival [18]. A study in China analyzing lymphocyte subsets in 507 patients with COVID-19 also found that among 341 patients with COVID-19, the counts of T-lymphocytes, CD4+ T-lymphocytes, and CD8+ T-lymphocytes were significantly lower in the deceased group than in the recovered group, and that cellular immunity was more impaired in patients with severe and critical disease than in patients with mild disease [19]. Age also becomes one of the risk factors for COVID-19 [20]. Levin et al. found that age is related to the infection fatality rate (IFR) of COVID-19. The age-specific IFR is very low in young people and children but gradually increases to 0.4% at 55 years, 1.4% at 65 years, 4.6% at 75 years, and 15% at 85 years [21].

Initial T-lymphocytes express CD45RA, and memory T-lymphocytes express CD45RO. In the peripheral blood, CD45RA+ T cells can be converted to CD45RO+ T-lymphocytes after antigen activation, and CD45RA and CD45RO are expressed differently in viral infections and autoimmune diseases [22, 23]. CD45RA+ T-lymphocytes and CD45RO+ T-lymphocytes are altered in patients with lymphoma [24], chronic hepatitis C [25], and systemic lupus erythematosus [23]. After a series of antiviral treatments in patients with chronic hepatitis C, the percentage of CD45RA+ T-lymphocytes decreased and CD45RO+ T-lymphocytes increased in patients, and it is thought that this change is related to the immunomodulation of the body during antiviral treatment [25]. Cytomegalovirus infection may contribute by driving CD8+ T-lymphocyte differentiation and inducing premature immune senescence and may lead to higher levels of chronic subclinical inflammation [26, 27]. Kern et al. demonstrated that cytomegalovirus infection results in a decrease in the number of CD8+ initial T-lymphocyte pool cells but an increase in the number of CD8+ effector T-lymphocytes [28].

CD28+ T-lymphocytes are functional, whereas CD28− T-lymphocytes are considered to be suppressor cells. When the expression of CD28 on the surface of T-lymphocytes is disturbed, the dynamic balance between immune activation and immune tolerance may be disrupted, further inducing autoimmune diseases [29]. The results are summarized as follows. Some studies have found that the proportion of CD4 + CD28+ T-lymphocytes decreases with HIV progression, and thus CD4 + CD28+ T-lymphocytes may be a marker for assessing HIV progression [30]. Regulatory T-lymphocytes (Tregs) play a key role in maintaining immune tolerance and modulating the immune response [31, 32]. Tregs under physiological conditions regulate host immune homeostasis and eliminate excessive host immune responses, which also favors the persistence of viral infections [31]. Tregs are associated with suppression of T cell immune responses in viral and bacterial infections [33]. Increased numbers of Tregs in patients with chronic hepatitis B (CHB) [34] and hepatitis C [35] viral infections impair the immune response to hepatitis B and C viruses, leading to viral persistence and chronic infection. Studies have shown that circulating and liver-resident Tregs positively influence the antiviral immune response and disease progression in patients with HBV [36]. In patients with CHB, the frequency of Tregs is usually increased and returns to normal levels in CHB patients after effective antiviral therapy [37, 38]. Upregulation of Tregs in CHB inhibits the activity and proliferation of HBV-specific CD4+ and CD8+ T cells and suppresses the secretion of IL-2 and IFN-γ, resulting in immunosuppression, reducing the ability of the host to clear increased HBV load [39]. During TDF treatment, a positive correlation between serum ALT levels and Treg frequency is observed only in HBeAg SC subjects, further confirming that a reduction in Treg frequency is associated with the course of HBeAg SC [40, 41].

The study of changes in the number of T-lymphocytes in the peripheral blood of COVID-19 patients is crucial for understanding COVID-19, and changes in their absolute counts play a suggestive role in immune function. Due to problems, such as the lack of widespread clinical lymphocyte subset testing, and the lagging development of flow cytometry in some medical institutions, most studies of T-lymphocytes in patients with COVID-19 have been limited to the detection of CD3+ T-lymphocytes, CD4+ T-lymphocytes, CD8+ T-lymphocytes, and the CD4+/CD8+ ratio [42]. However, the in-depth classification of T-lymphocytes can more accurately reflect the changes in the body's immune function and better help clinicians to assess the body's cellular immune function status. Therefore, it is important to investigate the changes of T-lymphocytes and their subpopulations in order to assess the condition and progression of COVID-19 and to take effective measures for the early intervention and treatment of COVID-19 in order to minimize its morbidity and mortality. In this study, T-lymphocytes were categorized into Th cells (CD3 + CD4+), Tregs (CD3 + CD4 + CD25+), naïve CD4+ T-lymphocytes (CD3 + CD4 + CD45RA+), CD4+ memory T-lymphocytes (CD3 + CD4 + CD45RO+) and their functional subpopulations (CD4 + CD28+), Tc cells (CD3 + CD8+), naïve CD8+ T-lymphocytes (CD3 + CD8 + CD45RA+), CD8+ memory T-lymphocytes (CD3 + CD8 + CD45RO+), and functional subpopulations of CD8+ T-lymphocytes (CD8 + CD28+). The results of COVID-19 patients were compared with those of healthy controls who underwent physical examination during the same period to analyze whether there were any differences between the two groups and to explore the correlation between the absolute and relative counts of each subpopulation of T-lymphocytes in patients with COVID-19 and the age of the patients, and to further study the relationship between absolute and relative counts of T-lymphocyte subpopulations and the severity of the disease.

## 2. Materials and Methods

### 2.1. Sample Collection

The diagnostic criteria and clinical classification of COVID-19 were based on the Diagnostic and Treatment Protocol for Novel Coronavirus Pneumonia (Trial Version 7) [43]. Sixty patients diagnosed with COVID-19 and admitted to the Second Affiliated Hospital of Shantou University Medical College between December 2022 and March 2023 were enrolled as the experimental group. Among them, 24 were male and 36 were female, with an age range of 7–94 years. The median age was 65 years (interquartile range: 53–73 years). Patients in the experimental group were categorized into the following clinical types: mild (13 cases), moderate (15 cases), severe (13 cases), and critical (19 cases). The control group consisted of 36 healthy individuals who underwent physical examinations during the same period. This group included 12 males and 24 females, with an age range of 6–88 years and a median age of 58 years (interquartile range: 45–64 years).

Exclusion criteria included the following:

1. Presence of other viral infections.
2. Immunodeficiency or autoimmune diseases.
3. Malignancy, current radiotherapy, or treatment with immunosuppressants.

The study was approved by the Medical Ethics Committee of the Second Affiliated Hospital of Shantou University Medical College (Approval no. 2023-42). We have obtained written consent from all participants, as well as consent from the parents/guardians of participants under the age of 16.

### 2.2. Detection of T-Lymphocytes and Their Subpopulations

The detection protocol was established by the FCSADiva software of the BD FACSCanto II flow cytometer (all fluorescent antibodies, hemolysates, and absolute counting tubes were produced by BD, USA). Antibody combinations used were as follows:

• FITC-CD3/PE-CD28/PerCP-CD45/PE-Cy7-CD25/APC-CD8/APC-Cy7-CD4.
• FITC-CD45RA/PE-CD45RO/PerCP-CD45/PE-Cy7-CD8/APC-CD3/APC-Cy7-CD4.

Isotype control tubes: FITC-γ/PE.

Three BD Trucount absolute counting tubes labeled No. 1, No. 2, and No. 3 were used. Approximately 18 μL of the mixed monoclonal fluorescent antibody cocktail was added to the bottom of each tube. Then, 50 μL of well-mixed EDTA-K2 anticoagulated peripheral blood was added to each tube. The tubes were gently shaken to mix thoroughly and incubated at room temperature for 15–20 min.

Next, 2 mL of hemolysin was added to each tube. The contents were mixed immediately at low speed and allowed to stand at room temperature, protected from light, for 10–12 min.

After hemolysis, 1 mL of the phosphate-buffered saline (PBS) was added to each tube. The samples were gently mixed, centrifuged at 500 × g for 5 min, and the supernatant was discarded. The cell pellet was retained and resuspended in 1 mL of PBS per tube.

Before sample acquisition, the optical and fluidic systems of the flow cytometer were calibrated, ensuring a coefficient of variation (CV) within 2%. Isotype control tubes were used to define negative and positive staining. Instrument voltages and compensation were adjusted to optimal settings.

A total of 50,000 cells were collected per tube. Samples were tested immediately using flow cytometry. The percentage of nucleated cells within the gated region was determined, and based on the number of microspheres in the Trucount tube, the absolute count of each cell subpopulation was calculated.

### 2.3. Statistical Analysis

Data were analyzed using SPSS Version 26.0. The Kruskal–Wallis test was used for nonparametric comparisons, and correlations were assessed using Spearman's rank correlation test. Graphs were generated using GraphPad Prism Version 8.0. A *p* value of less than 0.05 was considered statistically significant.

## 3. Results

### 3.1. Patients With New Coronary Arthritis Have Reduced Absolute Counts of T-Lymphocytes in Their Peripheral Blood, With a Disproportionate Number of Cells in Each Subpopulation

**Figure 1:**
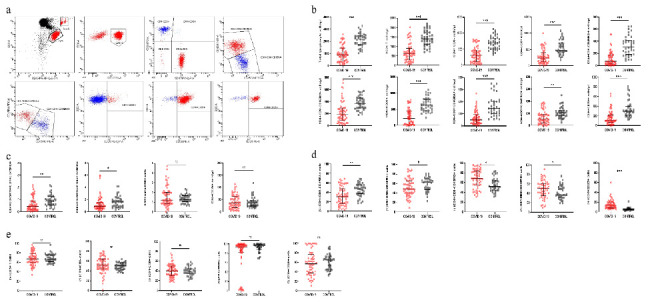
T-lymphocyte typing in patients with COVID-19, and distribution of lymphocytes and their subpopulations in patients and healthy controls. (a) Flow cytometric scatter plots of lymphocyte in-depth characterization. (b) Peripheral blood lymphocytes, T-lymphocytes, Th cells, Tc cells, naïve CD4+ T-lymphocytes, CD4+ memory T-lymphocytes, CD4+ T-lymphocyte functional subpopulations, naïve CD8+ T-lymphocytes, CD8+ memory T-lymphocytes, and CD8+ T-lymphocyte functional subpopulations in absolute counts. (c) CD4+ CD45RA+/CD4+ CD45RO+ ratio, CD8+ CD45RA+/CD8+ CD45RO+ ratio, CD4/CD8 ratio, and absolute regulatory T-lymphocyte counts. (d) Relative counts of CD4+ initial T-lymphocytes, CD8+ initial T-lymphocytes, CD4+ memory T-lymphocytes, CD8+ memory T-lymphocytes, and regulatory T-lymphocytes in the neoconjugate pneumonia group and the healthy control group. (e) Relative counts of T-lymphocytes, Th cells, Tc cells, CD4+ T-lymphocyte functional subsets, and relative counts of functional subpopulations of CD8+ T-lymphocytes. Dots show individual measurements for groups: COVID-19 group (red) and healthy control group (black); the black line shows the mean ± SD for each group, ^∗^*p* < 0.05, ^∗∗^*p* < 0.01, and ^∗∗∗^*p* < 0.001; ns: not statistically significant.

Flow cytometry was used to collect and analyze 50,000 cells, and CD45-SSC was used to set the gate analysis to calculate the number and percentage of circle-gated cells, and the absolute and relative counts of each subpopulation of cells were calculated based on the number of microspheres in the absolute counting tubes (Figure 1(a)). The results showed that the absolute counts of lymphocytes, T-lymphocytes, Th cells, Tc cells, naïve CD4+ T-lymphocytes, CD4+ memory T-lymphocytes, CD4+ T-lymphocyte functional subpopulations, naive CD8+ T-lymphocytes, CD8+ memory T-lymphocytes, and CD8+ T-lymphocyte functional subpopulations of peripheral blood lymphocytes in new conjugated pneumonia patients were lower compared to healthy individuals (Figure 1(b)). In addition, the CD4 + CD45RA+/CD4 + CD45RO+ and CD8 + CD45RA+/CD8 + CD45RO + ratios were reduced compared to healthy controls, but the CD4/CD8 ratio and absolute counts of Tregs in the peripheral blood of COVID-19 patients did not show statistically significant differences compared to those of the healthy controls (Figure 1(c)). The relative counts of naïve CD4+ and naïve CD8+ T-lymphocytes were also reduced in patients in the COVID-19 group compared to healthy controls, whereas the relative counts of CD4+ memory T-lymphocytes, CD8+ memory T-lymphocytes, and Tregs were elevated in patients compared to healthy controls (Figure 1(d)). However, the relative counts of T-lymphocytes, Th cells, Tc cells, CD4+ T-lymphocyte functional subsets, and CD8+ T-lymphocyte functional subsets were not statistically different compared to healthy controls (Figure 1(e)).

### 3.2. Absolute Counts of Peripheral Blood T-Lymphocytes and Their Subpopulations Are Lower in Critically Ill Patients Relative to Mild Patients

**Figure 2:**
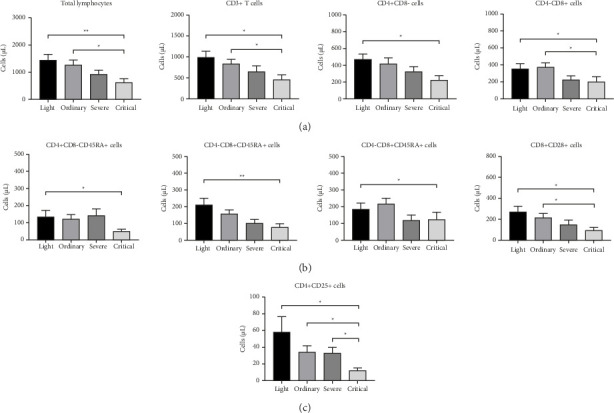
Distribution of peripheral blood lymphocytes and their subpopulations in patients with mild, normal, severe, and critical COVID-19. (a) Absolute counts of lymphocytes, T-lymphocytes, Th cells, and Tc cells in patients with COVID-19. (b) Absolute counts of naïve CD4+ T-lymphocytes, naïve CD8+ T-lymphocytes, CD8+ memory T-lymphocytes, and functional subpopulations of CD8+ T-lymphocytes. (c) Absolute counts of regulatory T-lymphocytes. Each bar graph represents the measurements of one group, with the height of each bar representing the mean, and the black line indicating the + SEM. ^∗^*p* < 0.05 and ^∗∗^*p* < 0.01. Unannotated indicates that the difference is not statistically significant.

Lymphocyte, T-lymphocyte, Th cell, Ts cell, naïve CD4+ T-lymphocyte, naïve CD8+ T-lymphocyte, CD8+ memory T-lymphocyte, regulatory T-lymphocyte, and CD8+ T-lymphocyte subpopulations of patients with COVID-19 pneumonia were statistically different in the comparison of the differences between the mild, moderate, severe, and critical groups, while the absolute counts, the CD4+ memory T-lymphocytes, the absolute counts of CD4+ T-lymphocyte functional subpopulations, CD4+/CD8+ ratio, CD4 + CD45RA+/CD4 + CD45RO + ratio, and CD8 + CD45RA+/CD8 + CD45RO + ratio were not statistically different between the four groups of mild, moderate, severe, and critical types (Table 1). The absolute counts of lymphocytes, T-lymphocytes, Ts cells, and CD8+ T-lymphocyte functional subpopulations were reduced in patients in the critical-type group compared to those in the mild and moderate groups (Figure 2(a)), and the absolute counts of Th cells, CD4+ initial T-lymphocytes, naïve CD8+ T-lymphocytes, and CD8+ memory T-lymphocytes were reduced in critical patients compared to those in the mild group (Figure 2(b)). Absolute counts of Tregs in patients in the critical group were reduced compared to those in the mild, moderate, and severe groups (Figure 2(c)). In terms of relative counts, there was no statistically significant difference between the four groups of patients in the mild, moderate, severe, and critical groups (results not shown).

### 3.3. Absolute Count of CD8+ Initial T-lymphocytes in the Peripheral Blood of the Patients in the New COVID-19 Pneumonia Group Was Negatively Correlated With the Age of the Patients

**Figure 3:**
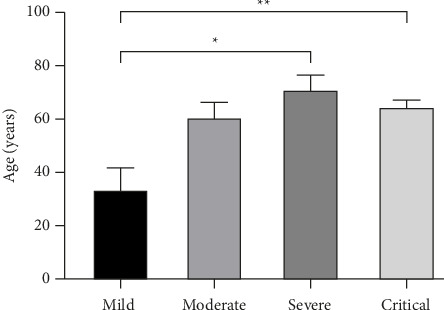
Age distribution of patients with mild, moderate, severe, and critical COVID-19. Each bar represents the measurements of one group, with the height of each bar representing the mean, and the black line indicating + SEM. ∗, *p* < 0.05; ∗∗, *p* < 0.01; unannotated indicates that the difference was not statistically significant.

We found that the absolute counts of naïve CD8+ T-lymphocytes in the peripheral blood of COVID-19 patients were negatively correlated with the age of the patients, i.e., the older the patients with COVID-19 the lower the absolute counts of CD8+ initial T-lymphocytes. The CD4 + CD45RA+/CD4 + CD45RO+ ratio and the CD8 + CD45RA+/CD8 + CD45RO+ ratio were also negatively correlated with the age of patients with COVID-19. Absolute counts of the remaining T-lymphocyte subpopulations did not correlate with the age of the patients (Table 2). No linear correlation was seen between relative counts and patient age (results not shown). In addition, patients in the severe and critical groups of *C. neoformans* pneumonia were older than those in the mild group (Figure 3).

## 4. Discussion

We show that total lymphocytes, T-lymphocytes, Th cells, and Tc cells are reduced in the peripheral blood of patients with COVID-19, with greater reductions observed in patients in the critical group compared to those in the mild and moderate groups, which is in agreement with the findings of others. Once a coronavirus-infected person loses a large number of CD4+ and CD8+ T-lymphocytes, the body's cellular immune function is in a very vulnerable situation. Elderly people have relatively weaker immunity and are more likely to develop severe or critical illness when infected with SARS-CoV-2. Serial testing of CD4+ T-lymphocytes, CD8+ T-lymphocytes, and their ratio in peripheral blood T-lymphocytes and their subpopulations in patients with COVID-19 helps us to understand the state of cellular immune function in patients.

In our study, we found that the absolute counts of naïve CD4+, CD4+ memory, naïve CD8+, and CD8+ memory T-lymphocytes in patients with COVID-19 are lower than those of healthy individuals, and the relative counts of naïve CD4+ T-lymphocytes, the relative counts of naïve CD8+ T-lymphocytes, the CD4 + CD45RA+/CD4 + CD45RO + ratio, and the CD8 + CD45RA+/CD8 + CD45RO + ratio are also lower than those of healthy controls, while the relative counts of CD4+ and CD8+ memory T-lymphocytes are elevated compared to those of healthy controls. Absolute counts of naïve CD4+ and naive CD8+ initial T-lymphocytes and CD8+ memory T-lymphocytes are decreased in patients in the critical group compared to patients in the mild group, and there was a negative correlation between the absolute counts of CD8+ initial T-lymphocytes, the CD4 + CD45RA+/CD4 + CD45RO+ ratio, and the CD8 + CD45RA+/CD8 + CD45RO+ ratio in the peripheral blood of patients with COVID-19 and the age of the patients. This might be due to the conversion of CD4 + CD45RA+ T-lymphocytes to CD4 + CD45RO+ T-lymphocytes after the invasion of the organism by the SARS-CoV-2, which facilitates a faster immune response. The decrease in the number of T-lymphocytes is associated with the degeneration of the thymus, which declines as the volume of the perivascular space of the human thymus increases with age. The decrease in the number of T-lymphocytes is related to the degeneration of the thymus. Thus, the number of initial T-lymphocytes detached from the thymus decreases dramatically with age [44]. The decrease in naïve CD8+ T-lymphocytes is greater compared to naïve CD4+ T-lymphocytes [45]. There is a negative correlation between the age of the patients and absolute naïve CD8+ T-lymphocyte counts, and as most of the patients with severe and critical illnesses are elderly, it is possible that the decrease in naive T-lymphocytes is also a factor in the decreased thymic generative capacity.

Our study shows that absolute counts of CD4+ T-lymphocyte functional subsets and CD8+ T-lymphocyte functional subsets are reduced in patients with COVID-19 compared to healthy controls, and the relative counts of Tregs are higher in patients with COVID-19, compared to healthy controls. Patients in the severe group had fewer absolute counts of CD4+ and CD8+ T-lymphocyte functional subsets than did those in the mild and moderate groups. The absolute counts of Tregs were all lower than those of patients in the mild, moderate, and severe groups. We hypothesized that the reduced numbers of CD4+ T-lymphocyte functional subsets and CD8+ T-lymphocyte functional subsets in patients with COVID-19 may be related to the overconsumption of CD4 + CD28+ T-lymphocytes and CD8 + CD28+ T-lymphocytes due to the attack of SARS-CoV-2 on the organism and/or to the dysfunction of the activation of T-lymphocytes caused by SARS-CoV-2 entry into the human body, and also to the dysfunction of the activation of CD4+ T-lymphocytes and CD8+ T-lymphocytes. When the body is invaded by SARS-CoV-2, a large number of immune cells are depleted, and the frequency of Tregs increases to regulate host immune homeostasis and eliminate the excessive host immune response.

## 5. Conclusions

In summary, SARS-CoV-2 infection has a significant effect on the number of T-lymphocytes and the ratio of their subpopulations, especially in critically ill and elderly patients, suggesting that SARS-CoV-2 infection severely affects the cellular immune function of the organism. We found that the absolute counts of T-lymphocytes and their subpopulations in the peripheral blood of patients with COVID-19 are reduced, and the proportion of each subpopulation was disproportionate, the absolute counts of T-lymphocytes and their subpopulations in the peripheral blood of critically ill patients are lower than those of mildly ill patients, and the absolute CD8+ T-lymphocyte count in the peripheral blood of patients with COVID-19 is negatively correlated with the age of the patients. The in-depth characterization of T-lymphocytes could more accurately reflect the changes in the body's immune function and better help clinicians to assess the state of the body's cellular immune function.

## Figures and Tables

**Table 1:** Comparison of the differences between the mild, moderate, severe, and critical types of pneumonia patients.

Clustering	Clinical classification				*p* value
	Mild	Moderate	Severe	Critical	
Lymphocyte cells	1423.00 (953.50, 2256.00)	893.00 (747.00, 1948.00)	899.00 (479.00, 1164.00)	339.00 (264.00, 936.00)	0.002^∗∗^
CD3+ T cells	1114.00 (567.00, 1548.00)	681.00 (635.00, 1175.00)	620.00 (303.00, 785.50)	288.00 (157.00, 714.00)	0.006^∗∗^
CD4 + CD8− cells	467.00 (260.00, 750.50)	422.00 (168.00, 726.00)	323.00 (197.00, 423.50)	109.00 (54.00, 405.00)	0.017^∗^
CD4− CD8+ cells	289.00 (185.00, 660.50)	351.00 (250.00, 568.00)	176.00 (107.50, 331.50)	146.00 (52.00, 239.00)	0.005^∗∗^
CD4+/CD8+	1.16 (0.87, 2.13)	1.28 (0.80, 1.96)	1.77 (0.88, 2.39)	1.21 (0.67, 2.10)	0.856
CD4 + CD8− CD45RA + cells	132.00 (32.50, 315.00)	137.00 (49.00, 164.00)	141.00 (35.50, 194.50)	36.00 (11.00, 69.00)	0.021^∗^
CD4 + CD8− CD45RO+ cells	250.00 (168.50, 467.00)	273.00 (114.00, 494.00)	166.00 (116.50, 247.50)	77.00 (45.00, 259.00)	0.050
CD4− CD8+ CD45RA+ cells	178.00 (106.50, 387.00)	151.00 (100.00, 224.00)	84.00 (44.50, 149.50)	58.00 (31.00, 112.00)	0.004^∗∗^
CD4− CD8+ CD45RO+ cells	148.00 (77.00, 287.00)	184.00 (96.00, 340.00)	76.00 (45.00, 168.50)	87.00 (21.00, 145.00)	0.032^∗^
CD4+ CD25+ cells	32.00 (14.00, 56.00)	28.00 (10.00, 51.00)	29.00 (16.50, 53.00)	6.00 (3.00, 24.00)	0.004^∗∗^
CD4+ CD28+ cells	293.00 (83.50, 695.50)	358.00 (11.00,7 03.00)	273.00 (69.50, 396.50)	106.00 (46.00, 380.00)	0.532
CD8+ CD28+ cells	182.00 (109.50, 325.00)	199.00 (88.00, 363.00)	98.00 (74.00, 193.50)	84.00 (18.00, 166.00)	0.006^∗∗^
CD4+ CD45RA+/CD4+ CD45RO+ cells	0.56 (0.10, 1.75)	0.46 (0.26, 0.55)	0.62 (0.31, 1.35)	0.33 (0.22, 0.82)	0.377
CD8+ CD45RA+/CD8+ CD45RO+ cells	1.13 (0.94, 4.57)	0.89 (0.53, 1.48)	0.93 (0.61, 1.31)	1.41 (0.65, 2.33)	0.283

*Note:* Statistical difference at *p* < 0.05.

^∗^ *p* < 0.05.

^∗∗^ *p* < 0.01.

**Table 2:** Correlation between T-lymphocyte subsets and age in patients in the neocoronary pneumonia group.

Clustering	Age	
	*p* value	*r*
Lymphocyte cells	0.280	−0.142
CD3+ T cells	0.204	−0.166
CD3+ CD4+ cells	0.386	−0.114
CD3+ CD8+ cells	0.209	−0.165
CD4/CD8	0.469	0.095
CD4+ CD8− CD45RA+ cells	0.065	−0.240
CD4+ CD8− CD45RO+ cells	0.782	0.037
CD4- CD8+ CD45RA+ cells	0.016^∗^	−0.310
CD4- CD8+ CD45RO+ cells	0.863	0.023
CD4+ CD25+ cells	0.141	−0.192
CD4+ CD28+ cells	0.138	−0.194
CD8+ CD28+ cells	0.456	−0.098
CD4 + CD45RA+/CD4 + CD45RO+	0.027^∗^	−0.285
CD8 + CD45RA+/CD8 + CD45RO+	0.001^∗∗^	−0.401

*Note:* Statistically different at *p* < 0.05.

^∗^ *p* < 0.05.

^∗∗^ *p* < 0.01.

## Data Availability

The data that support the findings of this study are available on request from the corresponding author. The data are not publicly available due to ethical restrictions.
